# Education Research: Training of Neurologists in South East Asian Countries: A Cross-Sectional Survey on the Current Neurology Curriculum

**DOI:** 10.1212/NE9.0000000000200201

**Published:** 2025-03-07

**Authors:** Si-Lei Fong, Kheng-Seang Lim, Shih-Hui Lim, Fitri Octaviana, Thang Cong Tran, Minh-An Thuy Le, Nijasri Charnnarong Suwanwela, Norazieda Yassin, Somchit Vorachit, Chum Navuth, Joesephine Casanova Gutrierrez, Khine Yee Mon, Brigido Simao de Deus, Wan-Chung Law, Chong-Tin Tan

**Affiliations:** 1 Division of Neurology, Department of Medicine, Faculty of Medicine, Universiti Malaya, Kuala Lumpur, Malaysia;; 2 Department of Neurology, National Neuroscience Institute, Singapore, and Duke-NUS Medical School, National University of Singapore, Singapore;; 3 Neurology Department, Faculty of Medicine, Universitas Indonesia Cipto Mangunkusumo Hospital, Jakarta, Indonesia;; 4 Department of Neurology, University of Medicine and Pharmacy at Ho Chi Minh City, Vietnam;; 5 Division of Neurology, Chulalongkorn University, Bangkok, Thailand;; 6 Brunei Neuroscience Stroke and Rehabilitation Centre, Pantai Jerudong Specialist Centre, Brunei Darussalam;; 7 University of Health Sciences of Lao PDR, Vientiane, Laos;; 8 Division of Neurology, School of Medicine, University of Health Science of Cambodia, Phnom Penh, Cambodia;; 9 College of Medicine, University of the Philippines, and De la Salle University Medical Centre, Philippine General Hospital, Manila, Philippines;; 10 Department of Neurology, Yangon General Hospital, Myanmar;; 11 Guido Valadares National Hospital, Dili, Timor Leste; and; 12 Department of Medicine, Sarawak General Hospital, Malaysia.

## Abstract

**Background and Objectives:**

This study aims to describe the variations in neurology training pathways in all Association of Southeast Asian Nations (ASEAN) countries.

**Methods:**

A cross-sectional survey using questionnaires was conducted among the representatives of ASEAN countries from October 2023 to March 2024.

**Results:**

Neurology training programs are available in 9 of the 11 ASEAN countries except Timor Leste and Cambodia. Despite the growing number of neurologists, with a doubling of the neurologist-to-patient ratio in most countries in the past 2 decades, the neurologist density per 100,000 population remained low. Thailand, Singapore, and Brunei Darussalam have more than 1 neurologist per 100,000 population compared with 2007 when only Singapore and Brunei Darussalam had more than this ratio. In Cambodia, Lao People's Democratic Republic (PDR), Myanmar, and Timur Leste, although the number of neurologists has increased substantially, the ratio of neurologists remains low, with less than 1 in a million population in Myanmar, 1:625,000 population in Lao PDR, 1:526,000 population in Cambodia, and 1:430,000 in Timur Leste. The total duration of training from undergraduate to certified neurologist varies greatly because of compulsory internal medicine (IM) training and postinternship services. To enroll in neurology training, candidates in 4 countries (Brunei, Singapore, Malaysia, and Myanmar) must have completed IM as a prerequisite. Candidates from Thailand and Indonesia must fulfill their 2-year compulsory government or general practice service requirement before they are eligible for neurology training. After fulfilling the eligibility criteria to enter neurology training, the overall training duration ranges from 3 to 13 years. Malaysia and Myanmar are countries where candidates spend more than 10 years becoming certified neurologists.

**Discussion:**

The number of neurologists and the neurologist-to-patient ratio have improved since 2007 in ASEAN countries. Diverse neurology curricula and the variable duration to complete neurology training and subspecialty practice are the main challenges in improving neurology training in ASEAN countries.

## Introduction

The Association of Southeast Asian Nations (ASEAN) was established in 1967 with 11 member states comprising Brunei Darussalam, Cambodia, Indonesia, Lao People's Democratic Republic (PDR), Malaysia, Myanmar, Philippines, Singapore, Thailand, Timor Leste, and Vietnam.^1^ According to the ASEAN Statistical Yearbook (2023), the total population in 2022 is 671.7 million.^2^ Most countries (except Brunei Darussalam, Malaysia, Singapore, and Thailand) are lower-middle and low-income countries with restricted resources. On a global scale, high-income countries such as the United States have been experiencing a progressive decline in the number of general neurologists, resulting in a mismatch between a high volume of neurology patients and a low number of neurologists.^3,4^ This insufficiency in the neurologic workforce is also encountered in the Asian Oceanian (AO) region, as highlighted in a recent review of the professional practice and training in neurology in the AO region.^5^ The ratio of neurologists to population ranges from 1:14,000 to as low as 1:32 million, with 9 of the 18 countries surveyed having less than 1 neurologist per 100,000 population.^5^ The Global Burden Disorders for all neurologic disorders, which are 2 times higher in lower-middle and upper-middle-income countries, reflect the need for neurologists in these regions.^6^

Focusing on ASEAN countries, a review in 2007 highlighted several issues, including older age at enrollment into neurology training programs, insufficient funding, limited direct clinical responsibilities, and no guidelines or local consensus on certain neurologic diseases published in native languages in certain countries. These factors may affect the quantity and quality of the neurologists trained in each country through their national training programs.^7^ In the past 17 years, as the national population has expanded, strategies have been implemented to improve healthcare facilities and professional education. We aim to describe the current neurology training program and structure in ASEAN countries in comparison with the previous review in 2007.

## Methods

A cross-sectional survey using questionnaires was conducted among the representatives of ASEAN countries (Brunei Darussalam, Cambodia, Indonesia, Lao PDR, Malaysia, Myanmar, Philippines, Singapore, Thailand, Timor Leste, and Vietnam) from October 2023 to March 2024. This survey included all 11 ASEAN countries as part of a collaborative initiative by the ASEAN Neurological Association (ASNA).

### Questionnaire

The questionnaire was designed by an expert panel of 4 consultant neurologists (S.-L.F., K.S.L., S.-H.L., and C.T.T.). S.-L.F., K.S.L., and S.-H.L. specialize in epilepsy, and C.T.T. specializes in epilepsy and neuroimmunology. K.S.L., S.-H.L., and C.T.T. are the current and past presidents of the ASNA, and C.T.T. is the chief editor of the *Neurology Asia* journal. They have an in-depth understanding of regional training and challenges. This questionnaire was adopted and modified from the previous questionnaire in 2007.^7^ The questionnaire comprised 6 sections, including (1) general information on the national state of the neurologic workforce and training structure, (2) eligibility for neurology training, (3) details of neurology training structure from undergraduate to completion of neurology training, (4) details of neurology training program, tuition fee, and sponsorship for conferences or workshops, (5) accreditation of trainees, and (6) subspecialization in ASEAN countries.

### Data Collection

The survey was emailed to 1 or 2 representatives from each country in October 2023. The respondents were mostly the executive committee members of the national neurological societies or senior neurologists in each country with at least 10 years of neurology practice. The respondents were encouraged to provide as many details as possible and to seek clarifications from their peers and colleagues from other institutions to obtain a nationwide overall view of the training. The data collection was completed in March 2024, and follow-up emails were sent to explain certain parts of the survey as needed. A descriptive analysis was performed on receiving responses from the country's representatives.

### Standard Protocol Approvals and Registrations

This study is exempted from ethical board review because it involves only open-source data collection and does not involve personal data collection.

### Data Availability

The data not published within this article will be made available by request from any qualified investigator.

## Results

### Growth of Neurologist Workforce and Training Centers

**Figure 1:**
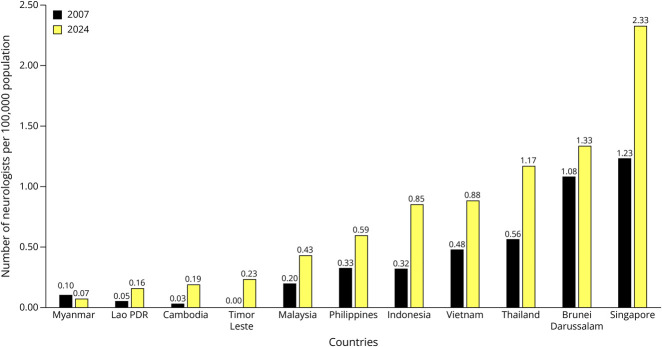
Number of Neurologists per 100,000 Population in ASEAN Countries in 2007 and 2024 ASEAN = Association of Southeast Asian Nations; PDR = People's Democratic Republic.

In the past 17 years, an increasing number of trained neurologists have been seen in all countries. Despite the growing number of neurologists, with a doubling of the neurologist-to-patient ratio in most countries, the neurologist density per 100,000 population remained low (Figure 1). Currently, only 3 of 11 countries (Thailand, Singapore, and Brunei Darussalam) have more than 1 neurologist per 100,000 population as compared with 2007 when only 2 countries (Singapore and Brunei Darussalam) had more than this ratio. In Cambodia, Lao PDR, Myanmar, and Timur Leste, although the number of neurologists has increased substantially, the ratio of neurologists remains low, with less than 1 in a million population in Myanmar, 1:625,000 population in Lao PDR, 1:526,000 population in Cambodia, and 1:430,000 in Timur Leste. All ASEAN countries (except Timor Leste) have their national neurology training programs. The national neurology training program is at the accreditation stage in Brunei Darussalam, and the national *Diplôme d’études spécialisées* curriculum in Cambodia will be finalized in 2024 (for resident acceptance in 2025). The number of neurology training centers in all countries except Singapore, Timor Leste, and Vietnam has increased over the years. For Singapore, there were 3 training programs in 2007, but 2 of the 3 had been reorganized into 1; thus, there are 2 training programs currently. The reduction of training centers in Vietnam resulted from a new national policy with a fixed neurology trainee enrollment quota in the universities due to a lack of postgraduate staff and the shutdown of some private medical schools. Despite the reduction of training centers, the number of trained neurologists in Vietnam increased over the years because the university enrolment quota was adjusted from 5–10 to 20–30 neurology trainees per institution per year. The Philippines has training centers dedicated to adult and pediatric neurology training. The number of neurology trainees enrolled per year varies across all ASEAN countries, ranging from 1 per year in Brunei Darussalam to 140 trainees in Vietnam (Table 1).

**Table 1:** Comparison of the Number of Neurologists, the Ratio of Neurologists to Population, Availability of National Neurology Training Program, Number of Neurology Training Centers, and Number of Neurology Trainees Enrolled Per Year Between 2007 and 2024

Year	Population (million)		No. of neurologists		Neurologist to population ratio^a^		Neurology training program		Neurology training center		Trainees enrolled per year^b^
	2007^c^	2024^d^	2007	2024	2007	2024	2007	2024	2007	2024	2024
Brunei Darussalam	0.37	0.45	4	6	1.08	1.33	N	Y^e^	0	1^e^	1
Cambodia	11.0	17.0	3	32	0.03	0.19	N	Y^f^	0	2^f^	5^f^
Indonesia	223.0	277.0	720	2,361	0.32	0.85	Y	Y	13	14	140–200
Lao PDR	5.9	7.7	3	12	0.05	0.16	N	Y	0	1	1–2^g^
Malaysia	25.0	32.7	50	140	0.20	0.43	Y	Y	4	16	12
Myanmar	10	54.7	10	39	0.10	0.07	Y	Y	1	4	1–11
Philippines	83.0	118.2	270	703	0.33	0.59	Y	Y	6	12 (A) 3 (P)	48–52
Singapore	4.3	6.0	53	140	1.23	2.33	Y	Y	3	2	6^h^
Timor Leste	0.8	1.3	0	3	0.00	0.23	N	N	0	0	0
Vietnam	84	98.8	402	870	0.48	0.88	Y	Y	12	7	140

Abbreviations: A = adults; ASEAN = Association of Southeast Asian Nations; P = pediatrics; PDR = People's Democratic Republic.

a Ratio of neurologist to 100,000 population.

b Data in 2007 were not available for comparison.

c Data as of 2007.

d Data as of 2023.

^e^Awaiting accreditation.

f National *Diplôme d’études spécialisées* curriculum was finalized in 2024 for resident acceptance in 2025.

g Last batch of local neurology trainees in 2023. A new fellowship program will be started in collaboration with other ASEAN countries.

h Six started in July 2024 (although there are 12–13 training positions per year).

### Prerequisite for Neurology Training

Each ASEAN country has its entrance criteria for enrolling trainees in national neurology training programs. Compulsory internal medicine (IM) qualification is an entrance criterion in 5 countries (Brunei Darussalam, Malaysia, Myanmar, Philippines (only for the Abridged Neurology Residency Program), and Singapore). Two of these countries (Malaysia and Myanmar) required a longer duration of IM training before neurology training in 2024, compared with 2007. Lao PDR had eliminated compulsory IM qualification as an entrance criterion. Of the 6 countries that do not require a mandatory IM qualification, other criteria such as completion of 1–3 years of government service (Thailand) and passing entrance examinations (Indonesia, Vietnam, Lao PDR) are required to qualify for neurology training. Cambodia is the only country that allows direct enrollment after completion of undergraduate. The compulsory IM training and its duration on enrollment into the neurology training program in each country is given in Table 2. The entrance criteria into the neurology training program are given in eTable 2.

**Table 2:** Compulsory IM Qualification and Duration of Training for Enrolment Into Neurology Training

Year	Compulsory IM qualification		Total duration of IM before neurology training (y)	
	2007	2024	2007	2024
Brunei Darussalam	Yes^a^	Yes^a^	3^a^	3^a^
Cambodia	N/A	No	N/A	0
Indonesia	No	No	0	0
Lao PDR	Yes	No	3	0
Malaysia	Yes	Yes	3	4.5–5
Myanmar	Yes	Yes	3	5
Philippines	Yes^b^	Yes^b^	0 in 5 centers, 0.5–1 in 1 center	0
Singapore	Yes	Yes	3^c^	3
Thailand	No	No	0	0
Timor Leste	N/A	N/A	N/A	N/A
Vietnam	No	No	0	0

Abbreviations: IM = internal medicine; MMed = Master of Medicine (IM); MMPI = Minnesota Multiphasic Personality Inventory; MRCP = Membership of Royal Colleges of Physicians; N/A = not available; TOFEL = Test of English as a Foreign Language; PDR = People's Democratic Republic.

a Only for lateral entry.

b Depending on enrolled neurology residency training program (not required for Straight Neurology Residency Program, at least 1 year in IM residency program for Abridged Neurology Residency Program).

c Previous IM-related clinical service postings before formal IM training posting were partially counted toward the 3-year IM training.

### Career Path From Undergraduate to a Certified Neurologist

**Figure 2:**
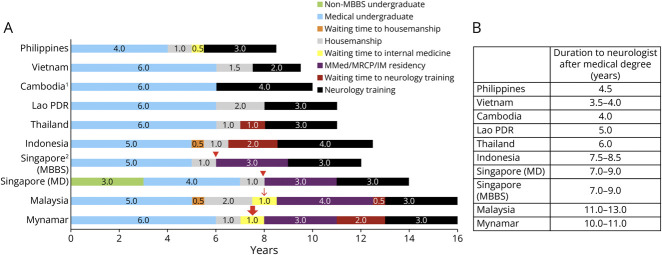
Duration of Neurology Training in ASEAN Countries (A) Minimum total training duration in ASEAN countries and duration spent in each stage before enrolment into the neurology training program. (B) Duration to become neurologist after medical degrees in ASEAN countries. ^1^Expected training duration based on the drafted National *Diplôme d’études spécialisées* curriculum. ^2^Additional 2 years for men for compulsory national service; waiting time ranges from 0 to several years depending on the success of IM residency application (red triangle arrows); minimum 1–2 years depending on acceptance into IM program (MMed or MRCP parallel pathway) (red line arrow); minimum 1–2 years before trial of entrance examination for MMed (red solid arrow). ASEAN = Association of Southeast Asian Nations; IM = internal medicine; MBBS = Bachelor of Medicine and Surgery; MD = Doctor of Medicine; MMed = Master of Medicine; MRCP = Membership of Royal Colleges of Physicians; PDR = People's Democratic Republic.

Four countries (Philippines, Vietnam, Cambodia, and Lao PDR) require a shorter duration (minimum 9–11 years, including undergraduate training) to become certified neurologists. Becoming a neurologist is the longest in Malaysia and Myanmar (minimum 16 years). The average duration of neurology training in most ASEAN countries is 3 years, except for Vietnam (2 years), Cambodia and Indonesia (4 years). In certain circumstances (such as maternity leave and sick leaves), the maximum allowed duration of neurology training is up to 3 years in Vietnam, 4 years in Myanmar and Malaysia, and 6 years in Indonesia. In the Philippines, there are 2 pathways to becoming a neurologist, either through the Straight Neurology Residency Program, which requires the clinician to have passed the physician licensure examination with a total of 3.5 years of training duration, while the Abridged Neurology Training Program requires at least 1-year experience in the IM residency program, giving a total duration of training of 3 years. Similarly in Singapore, there are 2 pathways to becoming medical doctors: a 5-year undergraduate pathway leading to a Bachelor of Medicine and Surgery or equivalent and another graduate pathway with any nonmedical degree plus 4 years in a graduate medical school, leading to a Doctor of Medicine degree. After 1 year of supervised clinical training after graduation from medical school (Housemanship, equivalent to the UK Foundation Program) and securing a training position, the maximum allowable training duration to be a neurologist in Singapore is 9 years. This includes 3 years of IM training and 3 years of Neurology training. Compulsory military service for men and maternity leave for women in Singapore are not counted toward these 9 years. The minimum and range of duration of neurology training in each ASEAN country are shown in Figure 2, A and B. Brunei Darussalam was not included in the figure because the program has not been accredited yet.

### Structure of Neurology Training

Most national neurology training programs included neurophysiology in the program. Other subspecialties such as neuroradiology, neuropathology, psychiatry, and acute stroke management are included in the training in most countries (eTable 1). All except Thailand and Myanmar trainees need to be rotated to other hospitals in their training. Lao PDR and Malaysia (Ministry of Health trainees) use a 2 + 1 training program where trainees spend the first 2 years in local hospitals and the third year in overseas institutions in Australia, Japan, the United Kingdom, the United States, Singapore, Taiwan, or Thailand for subspeciality training. Research output is compulsory in 5 of 11 countries (Thailand, Indonesia, Vietnam, Myanmar, and Cambodia). Five countries (Thailand, Indonesia, Vietnam, Myanmar, and the Philippines) have a structured outline of compulsory basic science training integrated into neurology training.

### Cost of Neurology Training Program

Three countries charge trainees tuition fees for the training, with Myanmar (US dollars [USD] 10 per month), Indonesia (USD 1,015–1,396 per semester), and Cambodia (USD 2,250 per academic year) (Table 3). In comparison with 2007, trainees in Vietnam may not need to pay the full tuition fees for neurology training. The funding for neurology trainees in Vietnam varies depending on the policy of each institution. It is largely influenced by the trainee's seniority and years of service commitment after graduation. This can lead to partial subsidies of up to 30% or even full waivers of tuition fees. Although no tuition fees are charged in Malaysia, trainees are required to serve a compulsory public service for 6 years after completing neurology training. They will be charged a penalty of at least USD 5,000 (before 2018) and a minimum of USD 105,000 since 2018 if the neurologists leave the public service within 6 years of completing their training. In most countries, there are conditional sponsorships for trainees to attend conferences or workshops, that is, funding would be provided if abstracts were submitted. Certain countries offer institutional sponsorships with quotas for the number of sponsored trainees (Indonesia) and annual funding (Singapore) (eTable 3).

**Table 3:** Neurology Training Structure and Tuition Fees

	Rotation to other hospital	Research	Basic science training	Fee
Brunei Darussalam	Yes	Optional	Optional	Free
Cambodia	Yes	Yes	Optional	USD 2,250 per academic year
Indonesia	Yes	Yes	Yes	USD 1,000–1,400 per semester per semester
Lao PDR	Yes^a^ (2 + 1 program)	Optional	Optional	Free
Malaysia	Yes (2 + 1 program)^b^	Optional	Optional	Free
Myanmar	Optional	Yes	Yes	USD 10 per month
Philippines	Yes	Optional	Yes^c^	Free
Singapore	Yes	Optional	Optional	Paid by the Ministry of Health
Thailand	Optional	Yes	Yes	Free
Vietnam	Yes	Yes	Yes	Free or partially subsidized

Abbreviations: PDR = People's Democratic Republic; USD = US dollars.

a Two years at Mitthaphab hospital and 1 year in France/any countries which accept the trainees.

b Only for Ministry of Health Hospitals, 2-year rotation in local hospitals and 1 year in an overseas institution.

c Neuroanatomy 30 hours per year, neurophysiology 16 hours per year, neurochemistry 12 hours per year, neuropathology 12 hours per year, and neuroradiology 12 hours per year.

### Additional Academic Obligations and Working Conditions

All ASEAN countries (except Myanmar) have integrated IM training into neurology training. Thailand and Lao PDR require 12 months of IM training, while Indonesia and Cambodia require 2–6 months, and Vietnam requires 1 month. Most trainees have teaching roles, but only those in Singapore, Myanmar, and the Ministry of Education in Malaysia have academic appointments. Leadership training is compulsory in the Philippines, while medical ethics training is compulsory in the Philippines and Singapore. The leadership program aims to train the trainee to be the future leader. In the Philippines, the trainees are part of the Philippines Neurological Association Junior Members Association where they are given responsibilities to organize training and projects (Table 4).

**Table 4:** Academic Responsibilities and Working Conditions During Neurology Training

	Integrated IM training (mo)	Teaching responsibilities	Academic appointments	Quality improvement/audit project	Medical ethics training^a^	Leadership training
Brunei Darussalam	12	Compulsory	None	Compulsory	Optional	Optional
Cambodia	3–6	Compulsory	None	None	Optional	Optional
Indonesia	2–3	Optional	None	None	Optional	Optional
Lao PDR	12	Compulsory	None	None	Optional	Optional
Malaysia	4	Compulsory	Compulsory^b^	None	Optional	Optional
Myanmar	0	Compulsory	Compulsory	None	Optional	Optional
Philippines	6	Compulsory	None	None	Compulsory	Compulsory
Singapore	6^c^	Compulsory	Compulsory^d^	Compulsory	Compulsory	Optional
Thailand	12	Compulsory	None	Compulsory	Optional	Optional
Vietnam	1	Compulsory	None	Compulsory	Optional	Optional

Abbreviation: IM = internal medicine; PDR = People's Democratic Republic.

^a^Good clinical practice, professionalism and/or health law.

b Only for trainees in university hospitals.

c Two months of internal medicine or geriatric medicine rotation per year for 3 years.

d Selected trainees with university teaching appointments.

### Assessment of Trainees and Accreditation of Neurology Training

Most ASEAN countries conduct annual formative assessments for neurology training. Vietnam, which had none in 2007, now requires miniclinical evaluations. The Philippines has expanded its formative assessment to include written, oral, and clinical components, beyond just oral examinations. Indonesia has also added more elements to its assessments. Each country has a national accreditation body to certify trainees on completing neurology training. Detailed information on the assessment and accreditation of neurology training in each country is given in eTable 4.

### Subspecialization in ASEAN Countries

**Figure 3:**
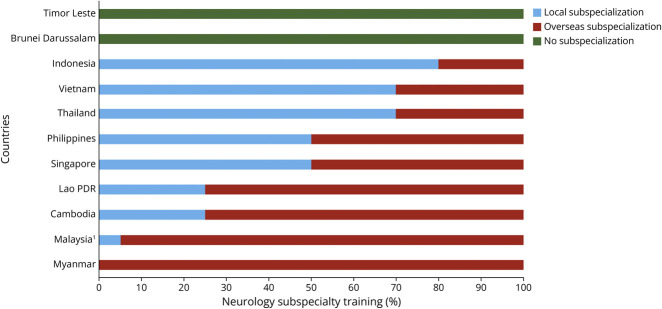
Location of Neurology Subspecialty Training ^1^The location of subspecialty training for trainees in the Ministry of Higher Education is based on personal preferences. PDR = People's Democratic Republic.

All neurologists in ASEAN countries except in Timor Leste and Brunei subspecialize. About 50% of neurologists practice in their subspecialized field more than 50% of the time in Singapore and Cambodia. Only 5%–25% of neurologists in other ASEAN countries (Thailand, Malaysia, Indonesia, Vietnam, and Myanmar) practice more than 50% of the time in their subspecialized fields because of the heavy general neurology workload (Figure 3).

## Discussion

In this review of the training of neurologists in ASEAN countries, we describe the diversity in the neurology curricula and duration of neurology training. Although the number of neurologists and the neurologist-to-patient ratio have improved since 2007 in ASEAN countries, striking a balance between producing a higher number of trained neurologists and the quality of their training and preparedness for clinical practice is challenging.

### Past and Present: Bird-Eye View of Neurology Training in ASEAN Countries

Neurology training in ASEAN countries has grown over the past 17 years. The issue of inadequate neurologists was highlighted in the previous survey in 2007.^7^ Up till 2018, the study which compiled data on neurology training worldwide published between 1994 and 2016, there were still a remarkable number of low- to upper-middle-income countries (11%–27%) surveyed that did not have neurologists.^8^ In our survey, although the neurologist-to-patient ratio remained low in most countries, we found that the number of neurologists in each country has increased over the years, and the neurologist-to-100,000 population ratio doubled in most countries. Notably, Singapore, Thailand, and Brunei Darussalam each have more than 1 neurologist for every 100,000 population. Brunei Darussalam, Cambodia, and Lao PDR have developed their national neurology training programs. The increased number of neurologists indicated that specialized neurology care would be more accessible to more populations in the country. However, there are significant regional variations in the density of neurologists in urban and rural areas within countries, in high- and lower-income countries. There were only 0.5% and 2.7% of neurologists practicing in rural areas in the United States and India, respectively, compared with cities.^9,10^ Neurologists tend to be drawn to urban areas due to better prospects in academic, research resources and professional opportunities. This could create a health care disparity between the urban and rural areas. From a health care professional perspective, the neurologist-to-patient ratio is lower in rural compared with urban areas and could easily lead to overwork of the neurologists. From a patient perspective, this disparity could cause delayed diagnosis and treatment for neurologic disorders that require subspecialized care.

In the previous World Federation of Neurology (WFN) survey in 2006, 8%–40% of neurology trainees from upper- and lower-middle-income countries were trained overseas. Ironically, only 27%–29% of the trainees returned to their home country to practice as neurologists after training.^11^ Before the introduction of the national neurology training program in Brunei Darussalam and Lao PDR, physicians interested in specializing in neurology were required to apply to overseas institutions recognized by the government, preferably those with a Memorandum of Understanding signed, such as Singapore and the United Kingdom. In Brunei Darussalam, these in-service training programs were funded by the government. In Lao PDR, the funding source varies according to the location of the host institutions. Training institutions in ASEAN countries would be funded by the government; otherwise, the trainees need to seek funding sources from other international organization that offer education fellowships. Establishing a national neurology training program would significantly reduce the extensive cost of sending trainees abroad for neurology training and prevent brain drain on completion of their neurology training at the same time. However, the diverse neurology training structures across ASEAN countries are worth discussing.

### Diverse Neurology Curriculum Across ASEAN Countries

#### Internal Medicine in Neurology Training

The main determinant of the variable neurology training duration across ASEAN countries is the prerequisite of completing IM training before enrolment. The total neurology training duration from completion of undergraduate to a certified neurologist was shortened (4.5–5 years) in countries that do not mandate IM training. In these countries, IM training was integrated into neurology training, with a duration ranging from 1 month (Vietnam) to a cumulative duration of 12 months (Lao PDR). A shorter total training duration allows the production of a greater number of neurologists over a shorter timeframe to meet the country's need to provide neurology specialist care to the population. In countries that include IM as a prerequisite for neurology training (Singapore, Malaysia, and Myanmar), the total duration of training to become a certified neurologist was longer (7–11 years). From a global perspective, this is similar to the observation that low- to upper-middle-income countries tend to have shorter duration of neurology training (average 3.5–4 years) compared with higher-income countries (up to 6 years).^12-16^ The European Neurological Society (ENS) survey also reported that 65% of European countries require compulsory general medical training between 0.5 and 2.5 years before neurology training.^15^

The need for IM services greatly influences the structure and content of neurology training programs and, therefore the diverse neurology curricula among ASEAN countries. In countries such as Singapore, Malaysia, and Myanmar, there is a higher demand for IM specialist services to ensure accessible health care services to suburban and rural populations. In such a health care structure, the competency in managing neurology-related and non–neurology-related IM issues is crucial. In Singapore, one of the main reasons to have 6 months of IM/geriatric medicine rotation during the 3-year neurology training is to maintain IM competency to ensure that neurologists can evaluate and manage their aging patients with IM-related comorbidities to minimize referrals to general physicians or other specialists.

The longer duration of neurology training is due to compulsory IM training, waiting periods between training stages, and mandatory military service for Singaporean men. Although these candidates may have stronger IM skills, they are at a more mature age when starting and completing neurology training. Financial and family commitments during training can be significant challenges.

Variable IM training as a prerequisite or integrated as part of neurology training is also seen in other regions of the world. In the United States, the Accreditation Council of Graduate Medical Education program for neurology requires 1-year IM training and 3-year general neurology training to be certified as a neurologist.^17^ In Europe, neurology training programs are organized differently across European countries.^3,15,18^ The ENS survey also reported that 65% of European countries required compulsory general medicine training ranging from 0.5 to 2.5 years before neurology training.^15^ While in the United Kingdom, an integrated 3-year IM and 2-year neurology training with a dual accreditation system is implemented.^3^ The training programs in Australia and New Zealand are similar to Singapore, Malaysia, and Myanmar where the trainees would need to complete a 3- to 5-year IM training, followed by a 2-year general neurology training and a 1-year general neurology elective or subspecialty fellowship, which altogether takes a minimum of 7 years to become a board-certified neurologist.

To address this long period of IM before entering neurology training, the consideration should ideally purely be based on the need of a neurologist to have a strong foundation of IM knowledge, for their lifelong professional practice as a neurologist. Two years of exposure to IM should be adequate for a clinician who wants to devote his career to neurology. One suspects that part of the reason why long years of IM is demanded is to fill the human resource needs of general physicians in the public health service by the authorities. The human resource need of internists should be solved in other ways and not at the expense of the development of neurology care.

#### Research and Basic Science Training

Countries such as Indonesia, Vietnam, and Cambodia required mandatory research projects and included basic science training as part of the neurology training, whereas they did not include IM as a prerequisite for enrolment. Research skills training can be part of the IM program or a 1-year research-based “B.Med.Sc” program or Master's in Clinical Investigation in Singapore. The training in research methodology is now part of the curriculum in undergraduate or graduate medical schools in Singapore. The training is crucial to equip neurologists with critical thinking and investigative skills, instead of just pattern recognition based on symptoms and signs; imaging and other neurophysiology tests; and treatment guidelines.

#### Tuition Fees and Sponsorship for Professional Education

As highlighted in the 2007 survey, trainees from Vietnam, Myanmar, and Indonesia need to pay tuition fees for their neurology training. As of 2024, Vietnam is now offering neurology training with partial to full subsidization of tuition fees. However, trainees' tuition fees are still required in Myanmar and Indonesia of which the tuition fee of USD 1,015 to 1,396 per semester is 25% of the Gross Domestic Product (GDP) per capita.^19^ In Cambodia, the payable tuition fee of USD 2,250 per academic year is 85% of the country's GDP per capita.^19^ In addition to the tuition fees, these trainees are often mature in age and also have family commitments. As a result, they might need to resort to locum to support themselves and their family financially. This may compromise the time they spend on neurology training. The training model of clinical neurology is often that of “apprenticeship,” and the trainees often also provide clinical services to the patients as part of their training. They should thus be paid for these services; the fees waived in lieu of the services they provide, so they can devote more time to study during the training. Compared with other countries included in the WFN survey in 2012, neurology trainees in Burkina Faso in West Africa (low-income country), Georgia, Jordan, and Macedonia (upper-middle-income countries) were also required to pay for their training.^12^ Up to 55% of neurology trainees in African countries who are not on the hospital payroll were required to pay for their training.^20^

Neurology trainees should be encouraged to attend conferences, workshops, and seminars to fill knowledge gaps and network. Most countries offer conditional sponsorships for these activities. In Indonesia, logistical limits restrict trainees from attending seminars for more than 2 days or twice during residency. Promoting quality workshops fosters lifelong learning.

### Assessment and Accreditation

Like the varied neurology curricula in ASEAN countries, the assessment and accreditation of neurologists also differ. In Lao PDR and Malaysia, trainees face no formal assessments, while other countries use different formats, from written examinations to comprehensive clinical assessments. Although simplified or no assessments reduce stress and offer flexibility, more detailed evaluations can provide structured learning and regular feedback from supervisors. Similarly, in European countries, although neurology residency training is accredited with reference based on the Global Standards of Quality Improvement in Medical Education (World Federation for Medical Education), accreditation varies among the countries.^18^

The accrediting boards for the neurologists who completed their training are independent in each ASEAN country. The final accreditation of the neurology trainees in countries such as Thailand involved at least 2 national boards. Formal assessment is usually based on familiarity with a basic set of knowledge. Formal assessment does help to drive learning to acquire this basic knowledge. However, it should not be overdone, at the expense of learning based on research and reading around the cases that the trainee must solve and manage. All in all, accreditation allows harmonization and an increase in mutual trust regionally or internationally. However, it could also obstruct innovation when all involved countries need to have a consensus whenever changes need to be made to keep up with the need for curriculum updates based on neurology advancement.

### Challenges in Subspecialization

Most neurology trainees in ASEAN countries receive subspecialty training abroad. In Singapore, trainees must complete general neurology training and practice for 2 years before being eligible for sponsorship for overseas subspecialty training. However, only 5%–25% of the neurologists in Thailand, Malaysia, Indonesia, Vietnam, and Myanmar can practice in their subspecialized fields >50% of the time. Subspecialization is driven by the advancement of neurology knowledge particularly in the diagnostic procedure and treatment in the fields of genetics, neuroimmunology, and radiology.^21^ However, there is a great challenge for subspecialized neurologists to practice in their fields due to a greater demand for general neurology care.

### Importance of English in Neurology Training

Importance of proficiency in English remains to be very important in the context of neurology training in South East Asia.^5^ The basic reason is that none of the local languages in South East Asia has a rich resource of knowledge on clinical neurology, and the availability of translation to local languages can cope with the rapid growth of neurology publications in English. The earlier comments that “the students are exposed to English early, and not burdened with too many foreign languages that they have to master” remains true.^5^

### Openness of Neurology Training in ASEAN Countries

Given the great diversity in neurology training across ASEAN countries, the challenge is daunting to improve the quality and quantity of neurologists trained in the region. In a previous study to determine the epilepsy research output in the South East Asia countries, it was found that “openness” and knowledge-based economy were the important factors determining the number of articles with an impact factor of more than 1.^22^ “Openness” was defined as whether a community is open to ideas and standards internationally. In the study, it was based on the use of English in medical schools and to access scientific literature, sending epilepsy fellows for overseas training, and requirements of overseas examiners in medical examinations and postgraduate thesis. “Openness” is also likely to be an important factor that determines the quality of neurology training in the region. Openness here is reflected in the teaching, assessment and accreditation, in-depth training or fellowship, and research and publication.^23^

#### Regional Training Collaboration

Establishing training collaboration among ASEAN countries is key to standardizing neurology training and ensuring consistent quality. This could involve sharing open educational resources, offering webinars led by regional experts (e.g., monthly ASNA webinars), and hosting joint workshops for hands-on training and networking among young neurologists across the region.

#### Regional Accreditation

Regional accreditation from undergraduate to postgraduate levels is crucial for producing quality specialists. Involving overseas examiners in regional institutions should be mandatory. The Asian Epilepsy Academy (ASEPA) EEG Certification Examination exemplifies how regional accreditation ensures neurologists meet standards. Such certifications can promote cross-recognition of qualifications and enhance regional collaboration.

#### Opportunities for Overseas Fellowship

The openness to international standards of fellowship training should be encouraged. All trainees should have equal access to information about funding opportunities and fellowship programs within or outside ASEAN countries, such as the ASNA Neurology Fellowship for clinical neurology attachment in ASEAN countries.^24^ Exchange programs for subspecialization could also improve their trained expertise and form a network of professional relationships and collaborative research opportunities across the region, such as epilepsy training fellowship under the ASEPA, International League Against Epilepsy (ILAE) AO Commission, movement disorders and clinical neurophysiology/neuromuscular fellowship program at the National Neurological Institute, and stroke fellowship in the National University Hospital in Singapore. The pooling of resources and expertise in ASEAN countries would also create a training environment that promotes excellence in each neurology subspecialty for better neurology care for the population in the region.

## Conclusion

The ASNA is the platform for the neurology fraternity to meet and communicate to develop neurology in this region. Throughout the years, the association has organized (1) biennial neurological association conferences since 1995, (2) established our regional neurology journal *Neurology Asia* to encourage research publications in the region, (3) joint ASEPA-ASNA EEG certification examinations, (4) regional workshops and symposiums on various subspecialty themes, (5) ASNA Neurology Fellowship to encourage general neurology attachment in ASEAN countries, and (6) monthly webinars on essential neurology topics by the field experts. The association continues to play a pivotal role in fostering collaboration, education, and the advancement of neurology within the ASEAN region, contributing to the overall growth of the field and nurturing more neurologists to meet the demand for neurology care in the region. The number of neurologists and the neurologist-to-patient ratio have improved over the years in ASEAN countries. Diverse neurology curricula, variable duration to complete neurology training, and subspecialty practice are the main challenges in improving neurology training in ASEAN countries.

## Glossary

AO: Asian Oceanian
ASEAN: Association of Southeast Asian Nations
ASEPA: Asian Epilepsy Academy
ASNA: ASEAN Neurological Association
ENS: European Neurological Society
GDP: Gross Domestic Product
ILAE: International League Against Epilepsy
IM: internal medicine
PDR: People's Democratic Republic
USD: US dollars
WFN: World Federation of Neurology
